# Supplementary material for: Mechanisms governing the pioneering and redistribution capabilities of the non-classical pioneer PU.1
Source: Nat Commun. 2020 Jan 21;11:402. doi: 10.1038/s41467-019-13960-2 (PMC6972792; doi:10.1038/s41467-019-13960-2)
Supplement: Supplementary file 7 — Source data [file 41467_2019_13960_MOESM7_ESM.zip › Source_Data/FigureS2/FigureS2B_MotifScanOutput/KmeansCluster4/homerResults/motif2.similar.html]

motif2

## Information for motif2

A
T
G
C
A
G
C
T
A
G
C
T
A
G
T
C
A
G
C
T
A
G
C
T
A
G
T
C
  
Reverse Opposite:  

C
T
A
G
T
C
G
A
C
T
G
A
A
C
T
G
C
T
G
A
C
T
G
A
A
T
C
G
  

|  |  |
| --- | --- |
| p-value: | 1e-620 |
| log p-value: | -1.428e+03 |
| Information Content per bp: | 1.747 |
| Number of Target Sequences with motif | 3337.0 |
| Percentage of Target Sequences with motif | 73.91% |
| Number of Background Sequences with motif | 13746.6 |
| Percentage of Background Sequences with motif | 32.89% |
| Average Position of motif in Targets | 99.4 +/- 49.1bp |
| Average Position of motif in Background | 100.6 +/- 84.7bp |
| Strand Bias (log2 ratio + to - strand density) | -0.1 |
| Multiplicity (# of sites on avg that occur together) | 1.71 |
| Motif File: | file (matrix) reverse opposite |

### Similar de novo motifs found

|  |  |  |  |  |  |  |  |
| --- | --- | --- | --- | --- | --- | --- | --- |
| Rank | Match Score | Redundant Motif | P-value | log P-value | % of Targets | % of Background | Motif file |
| 1 | 0.859 | C T A G T G C A T C A G A T C G G C T A T C G A A C T G T C G A C A T G | 1e-167 | -385.243134 | 29.41% | 12.75% | motif file (matrix) |
| 2 | 0.765 | A T G C A T G C A C G T A C G T A G T C A G T C A C T G A G T C | 1e-133 | -307.382066 | 24.92% | 10.96% | motif file (matrix) |
| 3 | 0.820 | C T A G A T G C C T G A A C T G C G T A C G T A A T C G A C G T | 1e-96 | -221.274911 | 22.06% | 10.60% | motif file (matrix) |
| 4 | 0.781 | C T A G C T G A C T A G C T A G C T G A C T A G T C A G C T G A C T A G C T A G C T G A T A C G | 1e-43 | -100.467394 | 4.98% | 1.52% | motif file (matrix) |
| 5 | 0.751 | T C A G C T G A A C T G C T A G T C G A C A T G T C A G T C G A C T A G C T A G T C G A | 1e-36 | -83.504096 | 2.99% | 0.69% | motif file (matrix) |
